# Functional assembly of surface microbiota of *Ulva fasciata* improves nutrient absorption efficiency and growth

**DOI:** 10.3389/fmicb.2024.1476073

**Published:** 2024-12-03

**Authors:** Han Wang, De-hua Li, Jing-ru Wang, Rong Wang, Chang-li Liang, Zhong Hu, Jun-he Liu

**Affiliations:** ^1^Biological and Food Engineering, Huanghuai University, Zhumadian, China; ^2^College of Computer and Artificial Intelligence, Huanghuai University, Zhumadian, China; ^3^Zhumadian Tobacco Company of Henan Province, Zhumadian, China; ^4^Department of Biology, Shantou University, Shantou, China

**Keywords:** *Ulva fasciata*, nitrogen concentration, function composition, synthetic community, nutrient efficiency

## Abstract

Macroalgae growth depends on biologically available nitrogen, such as ammonium and nitrate, making nitrogen the most common growth-limiting factor for macroalgae. However, the role of surface microorganisms in promoting nitrogen transformation and improving nitrogen utilization by macroalgae remains unclear. In this study, 228 bacterial strains were isolated from the surface of *U. fasciata*, and high-throughput sequencing revealed significant shifts in the composition of surface bacterial communities under different nitrogen concentrations. Key bacterial families such as Rhodobacteraceae and Flavobacteriaceae were identified as essential for nitrogen cycling. Network analysis indicated that Rhodobacteraceae and Flavobacteriaceae were central nodes in microbial interactions. A synthetic microbial community (SynCom2), comprising four strains, significantly increased the biomass, nitrogen, and phosphorus acquisition of *U. fasciata*, with soluble sugar, protein, and Chlorophyll *a* level increasing by 23.9–49.2%. Quantitative reverse transcription polymerase chain reaction (RT-qPCR) analysis revealed that compared to untreated control plants, SynCom2 enhanced the expression of key genes associated with photosynthesis (*rbcL*, 1.04-fold), lipid biosynthesis (*accD*, 11.21-fold), and growth hormone precursor pathways (*wrkY*, 9.54-fold). These findings suggest that SynCom2 promotes *U. fasciata* growth by improving nutrient acquisition and activating growth-related genes.

## Introduction

As the base of the marine food chain, algae play a critical role in supporting a variety of benthic animals. In addition to carbon dioxide and water, algae require nutrients like nitrogen and phosphorus to sustain their growth (Calatrava et al., 2024). Over time, algae have evolved to survive under various environmental conditions. For instance, *Ulva fasciata* (*U. fasciata*), a species of large green algae, thrives in nutrient-rich waters, leading to rapid biomass accumulation (Wang et al., 2024). While this growth can be advantageous in specific contexts, excessive proliferation of *U. fasciata* can harm coastal environments by disrupting ecosystem balance and influencing marine biogeochemical cycles (Zhang et al., 2015).

The growth and development of algae are influenced not only by abiotic factors but also by their interactions with associated microorganisms. Specific bacterial communities play a vital role in algal morphogenesis and nutrient acquisition (Egan et al., 2013; Wichard, 2015). To cope with fluctuating nutrient availability in marine environments, both algae and their associated microbes have developed strategies for nutrient acquisition and regulation (Ferranti and Delwiche, 2024; Li et al., 2019). Despite the long history of the algae-microbe relationship dating back to the earliest ecosystems (Wang et al., 2024), the mechanisms underlying these interactions remain poorly understood.

Algae release compounds that attract bacteria, creating suitable habitats for microbial colonization. These bacteria secrete substances that promote algal growth and morphogenesis (Ghaderiardakani et al., 2024). Studies have demonstrated that polysaccharides produced in co-culture environments can enhance this mutualistic relationship (Sial et al., 2020). Among the critical nutrients required by algae, nitrogen and phosphorus are mainly acquired using epiphytic bacteria residing on the algal surface (Pei et al., 2021). The structure and composition of these microbial communities directly influence the ability of algae to utilize nutrients (Smercina et al., 2019).

While numerous studies highlighted the importance of plant-associated microorganisms in nutrient acquisition, the complexity of microbial community structure has made it difficult to fully understand the role of beneficial bacteria (Li et al., 2018). These complex interactions can be mutualistic or competitive, affecting both community composition and the restructuring of microbial populations. Alterations in microbial community composition can, in turn, influence the growth and development of the host plant (Hu et al., 2016). By manipulating the abundance of specific beneficial bacteria, it may be possible to promote plant growth or address environmental concerns such as algal blooms (green tides).

Understanding the functional roles of epiphytic bacteria associated with *U. fasciata* is essential for addressing ecological challenges. In this study, *U. fasciata* was co-cultured with and without pure bacterial strains isolated from its surface to explore their roles in nitrogen uptake and plant growth. The results suggested that *U. fasciata* could modulate its epiphytic bacterial communities to adapt to varying nitrogen concentrations. Additionally, it was found that nitrogen availability for *U. fasciata* was largely dependent on these epiphytic bacteria, which work synergistically with the algae to absorb nitrogen and synthesize growth-promoting compounds. This study contributes to understanding how epiphytic bacteria aid *U. fasciata* in nutrient acquisition, laying the foundation for further exploration of plant-microbiome interactions and potential applications in promoting algal growth.

## Materials and methods

### Sample collection and *Ulva fasciata* cultivation

*Ulva fasciata* is a common wild species in southern China. In November 2022, *U. fasciata* was collected and transferred to a laboratory. The algae were washed several times with filtered seawater and placed in a light incubator containing artificial seawater (Berges et al., 2001) with 50 mmol/L (NO_3_^−^:NH_4_^+^ = 2:1) nitrogen. Artificial seawater was prepared following the method described by Berges et al. (2001), and all reagents were sourced from Sigma-Aldrich (St. Louis, MO, USA). *U. fasciata* was acclimated in artificial seawater for 1 week. The environmental parameters were as follows: temperature = 20 ± 1°C, light intensity = 800 lux (photoperiod L:D was 12 h:12 h), salinity = 30 PSU, and pH = 8.0.

Then, 20 g of *U. fasciata* was cultivated in nine closed plastic incubators of 28 × 27 × 18 cm. The nitrogen concentrations in each group in the three incubators were 10 (low concentration), 50 (control), and 200 mmol/L (high concentration). The nitrogen concentrations (10, 50, and 200 mmol/L) used in this experiment were selected based on ecological relevance and findings from previous studies on *Ulva* species. The control concentration of 50 mmol/L (NO_3_^−^:NH_4_^+^ = 2:1) was set following the protocol Berges et al. (2001) described, which simulates nitrogen levels commonly found in nutrient-enriched coastal waters. The low concentration of 10 mmol/L represents conditions with limited nitrogen availability, similar to those in oligotrophic environments, where nutrient limitation can affect algal growth (Li et al., 2019). The high concentration of 200 mmol/L was selected to simulate eutrophic conditions, where excessive nutrient input, especially nitrogen, can lead to algal blooms, such as green tides (Zhang et al., 2015). During the culture, the artificial seawater was replaced every 2 days. The experiment lasted 2 weeks in the same light incubator as the abovementioned conditions. Two grams of *U. fasciata* were sampled to determine the contents of soluble protein, sugar, phycocyanin, and Chlorophyll *a* (Schwenzfeier et al., 2011). Furthermore, seawater was taken to measure pH, salinity, dissolved oxygen, PO_4_^3−^, NO_2_^−^, NH_4_^+^, NO_3_^−^, and DSi_2_^+^ (Demirtas et al., 2008). Samples were collected at 7-time points (days 0, 2, 4, 6, 8, 10, and 12) and stored at −80°C for DNA extraction.

### DNA extraction and 16S rRNA sequencing

*Ulva fasciata* was kept in a 50-mL centrifuge tube, and an appropriate amount of sterile seawater was subsequently added. The samples were treated with ultrasound (1 min, 50 kHz, 5 s/5 s) to isolate bacteria from the surface of *U. fasciata*. After removing the *U. fasciata* with sterile tweezers, the sample was centrifuged at 4000 *g* for 10 min to collect the cell pallet, which was then used for bacterial DNA extraction. Total genomic DNA was extracted using the CTAB method (Zhou et al., 1996). All chemicals used for the CTAB extraction were purchased from Thermo Fisher Scientific Inc. (Waltham, MA, USA), and the procedure was carried out using an Eppendorf 5804R centrifuge (Eppendorf, Berlin, Germany). We used the PRISM^®^ 7500 rapid real-time PCR System to amplify high-quality DNA because it can achieve precise and efficient amplification of DNA in real-time monitoring (Huang et al., 2014). The PCR reactions used the TaKaRa Ex Taq™ DNA polymerase (TaKaRa Bio Inc., Shiga, Japan). The extracted DNA was sent to Shanghai Meiji Co., Ltd. (Shanghai, China) for high-throughput 16S rRNA measurement and epiphyte determination. Illumina MiSeq sequencing and universal primers 338F (5′-ACTCCTACGGGAGGCAGCAG-3′) and 806R (5′-GGACTACHVGGGTWTCTAAT-3′) were used to amplify the V3-V4 region of bacterial 16S rRNA gene.

### Bioinformatic analysis

After demultiplexing, sequence quality was assessed, with the removal of low-quality sequences using fastp (version 0.19.6) followed by merging with FLASH (version 1.2.11). The DADA2 plugin in the QIIME2 pipeline (version 2024) was then used with the recommended parameters for de-noising the sequences, yielding resolution at the single-nucleotide level according to the sample error profiles. The resulting amplicon sequence variants (ASVs) were annotated and classified using the SILVA 16S rRNA database (version 138) with the Vsearch consensus taxonomy classifier in QIIME2. To mitigate the impact of sequencing depth on diversity measures, the sequence count of individual samples was rarefied to 26,609, while maintaining an overall good coverage rate of 99.90%. Alpha diversity indices, including the observed ASVs and the Chao1, Shannon, and ACE indices, were calculated with Mothur (version 1.30.1). Similarities among the microbial communities in the different samples were assessed by principal coordinate analysis (PCoA) based on the Bray–Curtis dissimilarity using the vegan package in R (version 2.5–3). Monte Carlo permutation tests (9,999 permutations) were used for forward selection. In this analysis, the values of the *x*- and *y*-axes and the lengths of the corresponding arrows represented the significance of the different parameters in explaining taxon distributions across communities. Relationships within communities were explored by the construction of co-occurrence networks. These networks were analyzed using Cytoscape software, facilitating the visualization and exploration of complex interactions within the community. Spearman’s correlation coefficients were considered significant if above 0.6 or below −0.6, with *p* < 0.01.

### Taxonomic identification and phylogenetic analysis of potentially beneficial microorganisms

The surface of *U. fasciata* was gently scraped with a sterile knife, and the scraped materials were sequentially diluted and spread on the marine agar plate 2216E and placed in an incubator at 25°C for 3–4 days. Single colonies were selected, streaked, purified, and inoculated in marine broth 2216E. PCR amplification of the 16S rDNA gene of these strains isolated from the surface of *U. fasciata* was performed using 16S-27F (3′-AGAGTTTGATCCTGGCTCAG-5′) and 16S-1492R (3′-TACGGCTACCTTGTTACGACTT-5′) primers (Mincer et al., 2002). Then, the samples were sent to Bioengineering Co., Ltd. (Shanghai, China) for sequencing. These strains were cultured in marine broth 2216E until they reached the logarithmic growth phase. The bacterial suspension was mixed with 40% sterilized glycerol in a 1:1 ratio for cryopreservation, ensuring the viability of the bacteria during freezing and long-term storage. A total of 228 pure strains were isolated from the surface of *U. fasciata*. PCR amplification of the 16S rRNA gene of the isolated strains from *U. fasciata* was performed using the primers 16S-27F (3′-AGAGTTTGATCCTGGC TCAG-5′) and 16S-1492R (3′-TACGGCTACCTTGTTACGACTT-5′), following the protocol described by Mincer et al. (2002). The amplified 16S rDNA samples were sent to Bioengineering Co., Ltd. (Shanghai, China) for sequencing to enable strain identification and phylogenetic analysis. Given the predominant relative abundance of Rhodobacteraceae and Flavobacteriaceae within the colony, along with their strong correlation as illustrated in the network diagram, these taxa were selected for constructing an evolutionary tree utilizing the adjacency method implemented in MEGA 7.0 software.

### Screening potentially beneficial microbes

The N_2_ fixed marker gene (nifH) was confirmed by PCR assay using the following primer pairs: nifH-F (AAAGGYGGWATC GGYAARTCCACCAC) and nifH-R (TTGTTSGCSGCRTACATSG CCATCAT) (Rösch et al., 2002). As indole-3-acetic acid (IAA) regulates plant growth and development, we also tested the strains’ capacity to produce IAA (Ahemad and Kibret, 2014). Salkowski’s colorimetric method was used to screen for IAA production by strains according to Bric et al.’s (1991) research. The strains were cultured in 50 mL of 2,216 E medium at 25°C for 3 days, and the bacterial culture medium was centrifuged at 10000 rpm for 10 min at 4°C. The cell pellet was washed twice with phosphate-buffered saline (PBS), supplemented with 1 g/L of L-tryptophan, and then oscillated in an oscillating incubator for 7 days. After centrifugation, 1 mL of the supernatant was added to 2 mL of Salkowski reagent (1 mL of 0.5 mol/ L FeCl_3_ and 49 mL of 35% HClO_4_). The mixture was incubated in the dark at 25°C for 30 min, and the absorbance was measured at 530 nm using a spectrophotometer. The concentration of IAA was estimated using the standard IAA curve. All IAA measurements were performed in triplicate. To determine phosphorus solubilizing activity, Ca₃(PO₄)₂ was utilized as an insoluble inorganic phosphate source. Members of the Rhodobacteraceae and Flavobacteriaceae families that could activate and solubilize phosphate were screened. The strain was incubated at 25°C for 3 days, and then, the phosphorus solubilization capacity was screened in a solid NBRIP medium. In a 250-mL conical flask, 50 mL of NBRIP medium was mixed with 100 μL of strain culture at neutral initial pH, then incubated for 7 days in a shaker at 25°C, and repeated inoculation three times. The culture was centrifuged at 5000 rpm for 20 min. The precipitation of P_2_O_5_ was determined by the molybdenum-blue method (Galhardo and Masini, 2000).

### Construction of SynCom

The changes in bacterial abundance on the surface of *U. fasciata* under different nitrogen concentrations and the essential nodes in the network *indicated* that Rhodobacteraceae and Flavobacteriaceae may be potential bacterial strains to improve nutrient absorption efficiency and growth of *U. fasciata*. Therefore, 29 strains of Rhodobacteraceae and Flavobacteriaceae were combined into SynCom1. Based on the above functional screening, 5 strains with the *nifH* gene, high phosphorus solubilization capacity, and high IAA yield were selected from 29 strains as candidates of SynCom2. SynCom was co-cultured with sterilized *U. fasciata* without bacteria on the surface without nitrogen.

### Co-culture experiments without N addition

The preparation method of *U. fasciata* without surface bacteria (WSB *U. fasciata*) was summarized as follows: *U. fasciata* was collected from the field and transported back to the laboratory on the same day. The surface of *U. fasciata* was thrice washed with sterile seawater and cleaned with ultrasonic (10 min, 50 W, 5 s/5 s) to remove bacteria. Antibiotics, including streptomycin sulfate (final concentration of 100 μg/mL), amphotericin (0.25 μg/mL), ampicillin (100 μg/mL), and imidazole (0.1 μg/mL), were added and incubated for 4 days. The exact amount of antibiotics was added again on the third day. Next, a sterile cotton swab was dipped in a multi-enzyme cleaning solution (3 M) and used to quickly scrub the surface of *U. fasciata*. Then, *U. fasciata* was rinsed with a large volume of sterile seawater to obtain WSB *U. fasciata.* The water from the last rinse was applied to the solid medium 2216E and cultured for 1 week. The absence of bacteria was indicative of WSB *U. fasciata*. SynCom strain was activated at 25°C. After 3 days of activation, the cell pellet was collected by centrifugation (at 4000 × *g*) and twice washed with sterile seawater. It was, after that, resuspended to a concentration of 10^7^ strains per mL. After mixing equal volumes (1:1 ratio), the final volume in each SynCom group was adjusted to 5 mL. The mixed bacteria were added into an airtight bottle and co-cultured with WSB *U. fasciata* in artificial seawater without nitrogen addition. The treatment of WSB *U. fasciata* without adding bacteria served as the control. On the last day of the experiment (day 7), the co-cultured *U. fasciata* was washed with a large amount of sterile seawater, and the surface strains of *U. fasciata* were obtained by ultrasonic shock, which was coated in marine medium 2216E. The growth of the strain in the medium was the same as that initially added, indicating that the co-culture was unpolluted. During the experiment, each SynCom group was repeated at least three times.

### SynCom functional evaluation

To assess the effects of SynCom on the growth of *U. fasciata*, samples of *U. fasciata* with similar growth and size were collected from the same batch using a multi-spot sampling method. Surface moisture was absorbed using sterile filter paper, and the samples were weighed for wet weight. Three samples were dried in an oven at 60°C until they reached a constant dry weight, while the remaining six samples were sterilized for the co-culture experiment. After 1 week of co-culturing SynCom with WSB *U. fasciata*, the wet and dry weights of the *U. fasciata* samples were measured. The contents of soluble sugar, protein, phycocyanin, and Chlorophyll a were also determined. Nitrogen content in the samples was calculated according to the method described by Farina et al. (1991), while phosphorus concentration was determined using phosphomolybdenum blue spectrophotometry and a UV–vis spectrophotometer.

### Activity of glutamine synthetase (GS)

About 0.5 g *U. fasciata* sample (wet weight) was homogenized in liquid nitrogen with 5 mL of 0.2 M HEPES buffer (pH 7.9). The homogenate was centrifuged for 20 min, and the supernatant was used to determine GS activity based on the method proposed by Slawky and Rodier (1988).

### *Ulva fasciata* RNA extraction and RT-qPCR of some key genes

Total RNA of co-cultured *U. fasciata* was extracted by TRIzol reagent under low nitrogen conditions (Ma et al., 2011). The culture of uninoculated WSB *U. fasciata* was used as a negative control. DNA enzyme was used to remove possible DNA, and gel electrophoresis was used to confirm the DNA removal and ensure the RNA’s purity. RNA quantification was performed using a spectrophotometer, and RNA was used to synthesize the first strand of cDNA. The analysis was conducted in a 96-well plate using the SYBR Green method, and the *U. fasciata β*-actin gene served as an internal control. The effect of the synthetic community (SynCom) on *U. fasciata* at nitrogen-free concentration was confirmed by quantifying the expression levels of three key genes involved in photosynthesis (*rbcL*), lipid biosynthesis (*accD*), and growth hormone precursor (*wrkY*) pathways. The primer sequences used in this study are listed in Supplementary Table S1. Quantitative reverse transcription polymerase chain reaction (RT-qPCR) reactions were carried out at Each reaction was carried out in triplicate, and the 2^−△△CT^ formula was used to calculate the RT-qPCR amplification data.

## Results

### Changes in bacteria community composition during *Ulva fasciata* cultivation

**Figure 1:**
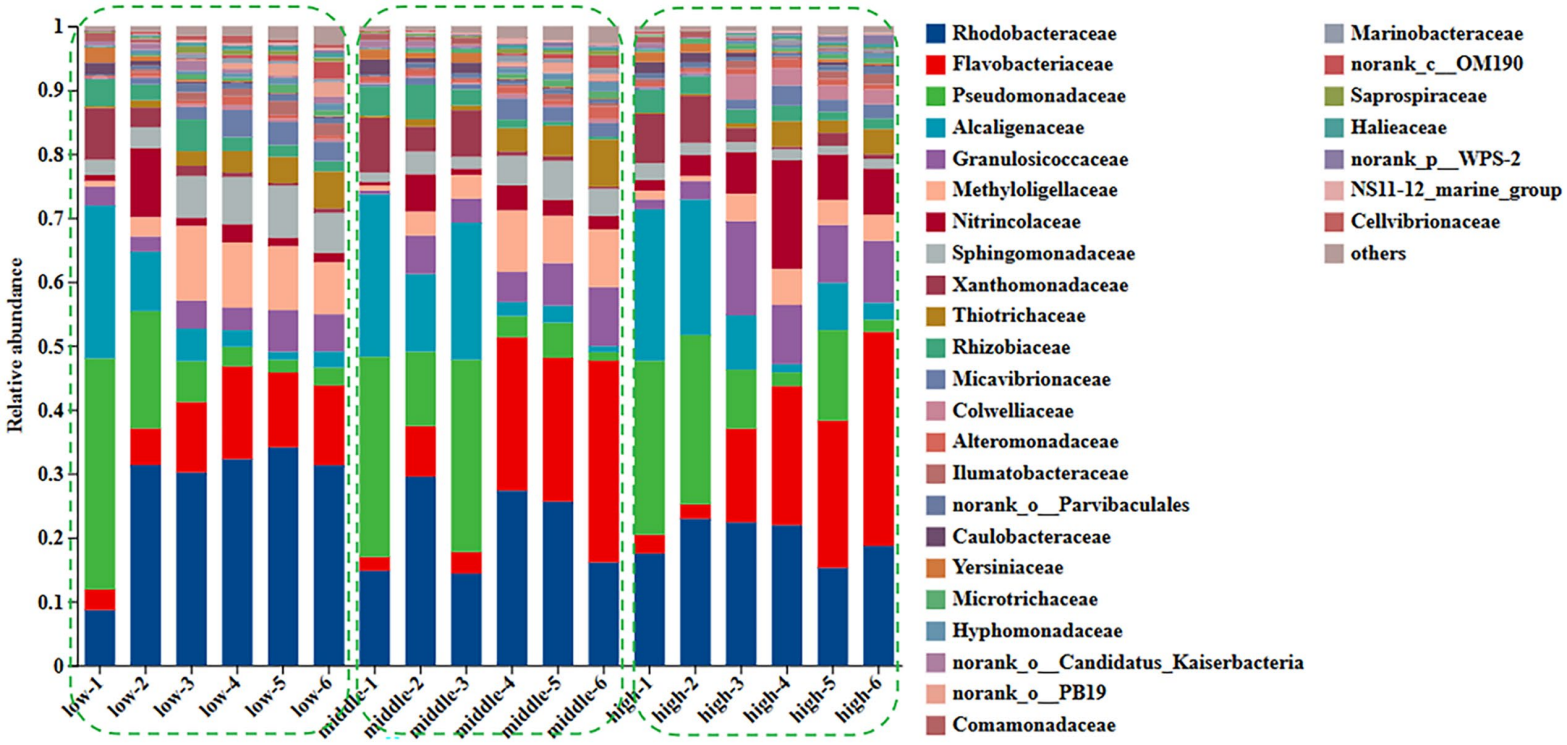
Changes of family level in surface community of *Ulva* under different nitrogen concentration.

A total of 2,741,338 high-quality sequences were obtained from *U. fasciata* samples (*n* = 54). The relative abundance of bacterial communities was analyzed at the genus level, and a Chi-square test was used (Figure 1). The results revealed that the bacterial community changed dramatically during the culture at low nitrogen concentration.

The relative abundance of the family Rhodobacteraceae (observed range: approximately 8.6–31.3%, *p* < 0.01) and Flavobacteriaceae (approximately 3.2–12.5%, *p* < 0.01) increased significantly, while the relative abundance of members of the families Pseudomonadaceae (decreasing from approximately 36.1 to 2.8%, *p* < 0.01) and Alcaligenaceae (decreasing from around 23.9 to 2.5%, *p* < 0.01) declined significantly during the growth of *U. fasciata* under low nitrogen levels. Under normal nitrogen levels, the relative abundance of Rhodobacteraceae (ranging from approximately 14.8 to 16.1%, *p* < 0.01) and Flavobacteriaceae (ranging from approximately 2.1 to 31.6%, *p* < 0.01) increased, whereas the relative abundance of Pseudomonadaceae (from 31.3 to 2.8%, *p* < 0.01) and Alcaligenaceae (from 25.4 to 1.3%, *p* < 0.01) showed a marked decrease. Similarly, when *U. fasciata* was cultured under high nitrogen concentrations, the relative abundance of Rhodobacteraceae increased from 17.5 to 18.7% (*p* < 0.01), and Flavobacteriaceae from 2.9 to 33.5% (*p* < 0.01). In contrast, the relative abundance of Pseudomonadaceae (decreasing from 27.2 to 18.9%, *p* < 0.01) and Alcaligenaceae (decreasing from 23.7 to 2.7%, *p* < 0.01) significantly declined. The relative abundance of Rhodobacteraceae likely reflects its direct involvement in nitrogen transformations, such as ammonification and nitrification, processes critical for regulating nitrogen availability within the ecosystem. Different forms of nitrogen, such as ammonium, nitrate, or organic nitrogen, might selectively influence the growth of Flavobacteriaceae, which could also be acting in concert with other nitrogen-cycling microbes to balance organic matter degradation and nutrient recycling. Under treatments with varying nitrogen levels, both microbial families may exhibit changes in abundance as a response to nitrogen limitation or surplus, adjusting their metabolic strategies to conserve or exploit available nitrogen sources for survival and growth (Figure 1).

**Figure 2:**
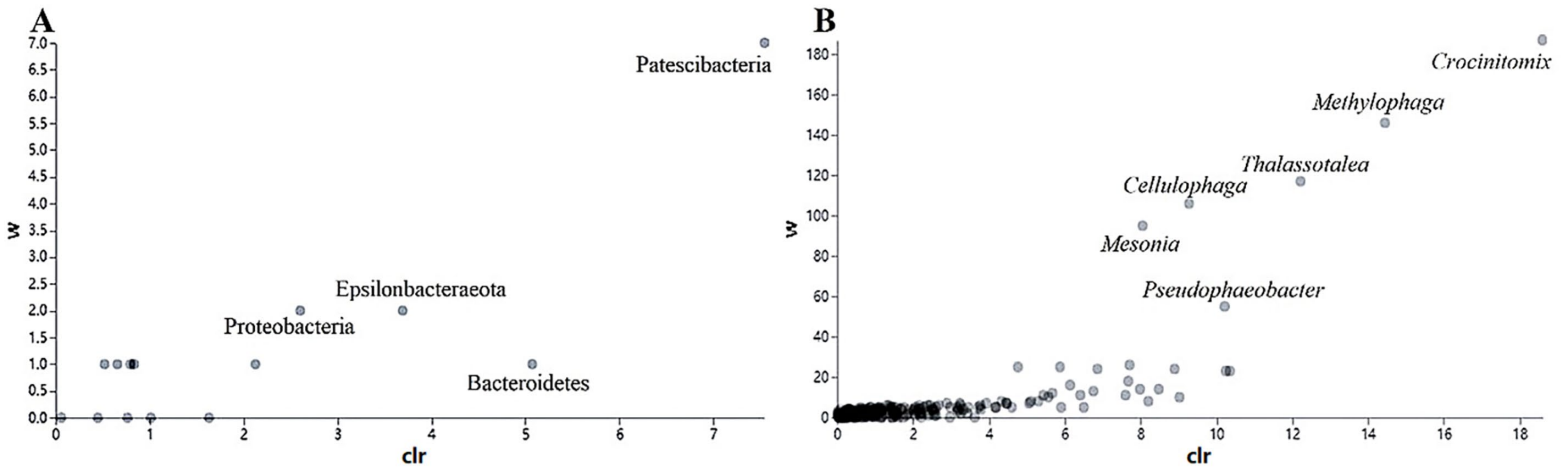
Used the ANCOM statistical analysis to calculate the relative abundance of different bacterial species at phylum **(A)** and genus **(B)** levels with different nitrogen concentration.

**Figure 3:**
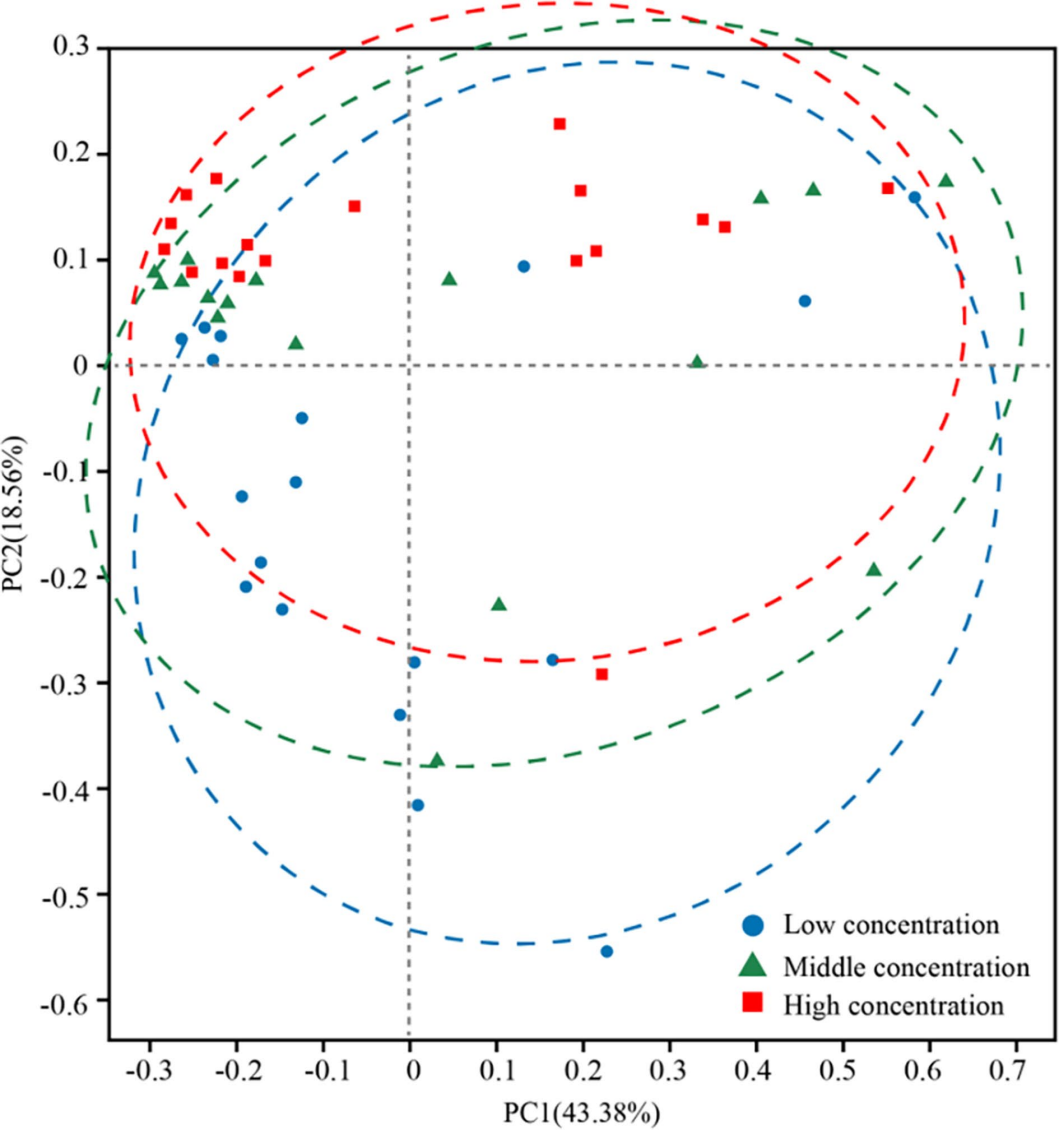
PCoA analysis under different nitrogen concentration treatment family level.

In addition, the abundance of Rhodobacteraceae was elevated during the culture of *U. fasciata* with the low nitrogen concentration, while it decreased at a high N concentration. In addition, ANCOM statistical analysis revealed that common nitrogen-fixing bacteria, such as *Methylophaga*, and members of Flavobacteriaceae (e.g., *Cellulophaga* and *Mesonia*) and Rhodobacteraceae (e.g., *Pseudophaeobacter*), exhibited significant differences under varying nitrogen conditions (Figure 2). Moreover, the PCoA based on Bray–Curtis dissimilarity revealed that high concentration of nitrogen had a greater effect on the bacterial community than its low concentration (Figure 3). The PCoA presented in this study accurately reflects the variation in microbial communities (or relevant metric) across different nitrogen concentration treatments. The first principal coordinate (PC1) accounted for 43.38% of the variation, while the second principal coordinate (PC2) explained 18.56%. These values indicate that the first two components capture a significant proportion of the variation in the dataset. The observed separation between the groups treated with different nitrogen concentrations suggests nitrogen plays a substantial role in shaping the community structure. The permutational multivariate analysis of variance (PERMANOVA) was conducted to assess this separation’s significance statistically, confirming that the differences between nitrogen treatment groups were significant (*p* = 0.01). The correlation between nitrogen concentration and PC1 was also calculated, showing a strong positive correlation (*r* = 0.78), further supporting nitrogen’s influence on the community variation. Shannon index demonstrated that the diversity of bacterial community harboring the surface of *U. fasciata* cultured at low nitrogen concentration was similar to that at high concentration (Table 1).

**Table 1:** Diversity index of surface microorganisms of *U. fasciata* at different nitrogen concentration.

Sample		Shannon	Ace	Chao 1
Low concentration	1	1.93 ± 0.42^a^	103.73 ± 8.54^a^	102.05 ± 18.78^a^
	2	2.44 ± 0.22^b^	103.96 ± 14.84^a^	94.34 ± 8.24^a^
	3	2.79 ± 0.18^b^	104.55 ± 9.79^a^	113.82 ± 24.72^a^
	4	2.77 ± 0.07^b^	104.72 ± 13.99^a^	109.92 ± 5.16^a^
	5	2.70 ± 0.15^b^	105.21 ± 8.80^a^	103.55 ± 8.83^a^
	6	2.85 ± 0.21^b^	106.26 ± 24.82^a^	120.95 ± 22.71^a^
Middle concentration	1	2.31 ± 0.51^b^	110.74 ± 22.97^a^	101.90 ± 22.00^a^
	2	2.15 ± 0.45^b^	110.79 ± 40.59^a^	100.16 ± 4.48^a^
	3	2.33 ± 0.40^b^	111.41 ± 10.57^a^	110.53 ± 8.46^a^
	4	2.71 ± 0.14^b^	116.76 ± 20.26^a^	133.07 ± 56.25^a^
	5	2.73 ± 0.15^b^	118.33 ± 14.21^a^	107.52 ± 23.31^a^
	6	2.66 ± 0.10^b^	135.91 ± 46.49^a^	101.13 ± 14.93^a^
High concentration	1	2.46 ± 0.08^b^	81.25 ± 3.06^a^	102.71 ± 13.64^a^
	2	2.22 ± 0.13^b^	86.08 ± 6.59^a^	98.95 ± 19.47^a^
	3	2.52 ± 0.03^b^	90.30 ± 11.79^a^	84.20 ± 7.34^a^
	4	2.40 ± 0.11^b^	98.31 ± 11.02^a^	82.33 ± 11.86^a^
	5	2.53 ± 0.17^b^	99.58 ± 15.90^a^	96.44 ± 16.95^a^
	6	2.45 ± 0.30^b^	99.72 ± 16.36^a^	86.64 ± 10.76^a^

The error bars are standard deviation; different letters on the error lines indicate significant differences between different treatments (α = 0.05).

### Environmental factors affect the bacterial community

**Figure 4:**
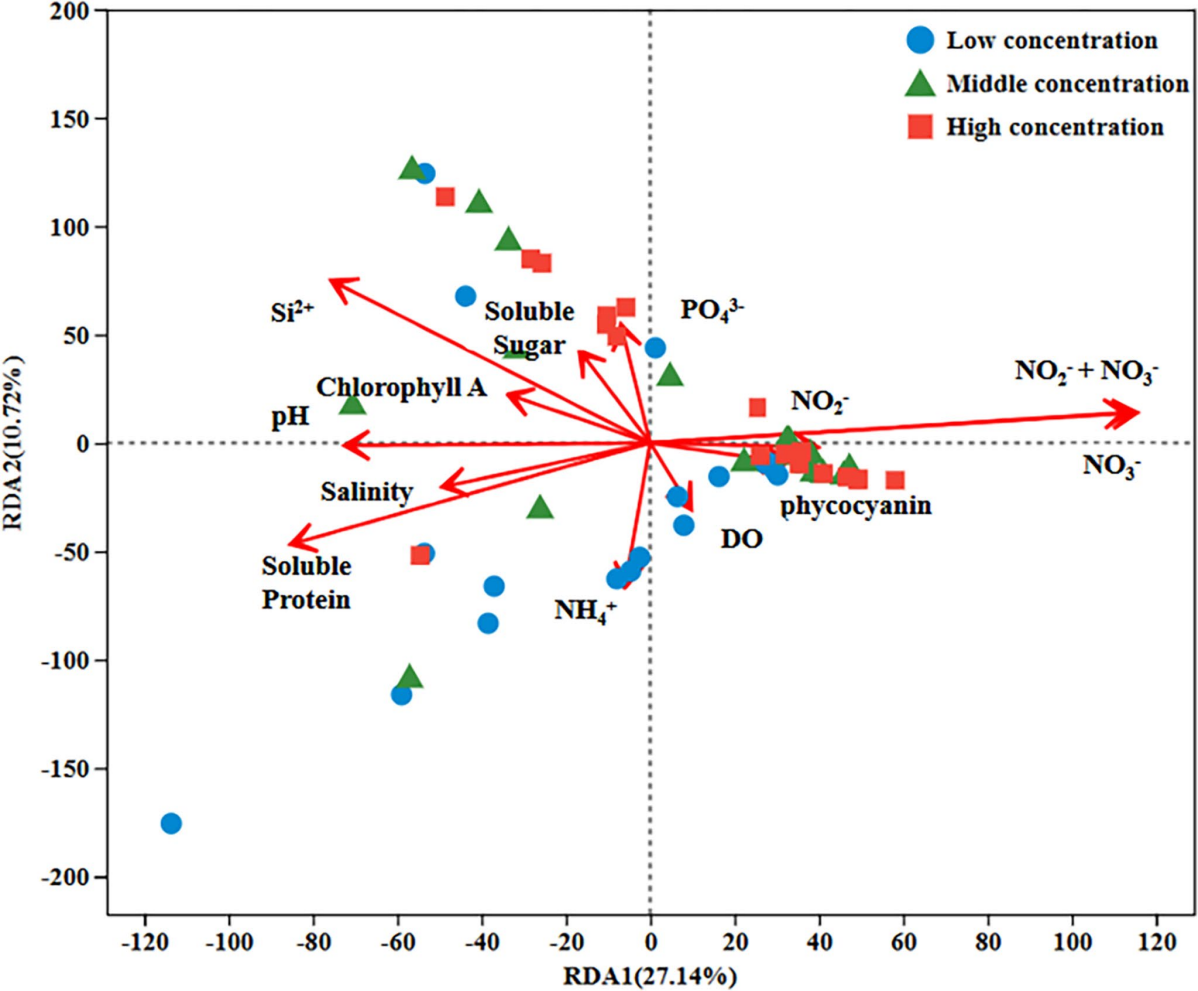
RDA analysis of environmental factors and bacterial genera under different nitrogen concentration.

RDA of environmental factors and microbiota was performed to identify the factors influencing changes in the bacterial community during the growth of *U. fasciata* (Figure 4). The results showed that the arrows representing NO_3_^−^ were the longest compared with other influential factors, in which NO_3_^−^ had a greater influence on the surface microbiota of *U. fasciata* cultured under different nitrogen concentrations. In this experiment, it was also found that DSi_2_^+^ had a greater impact on most microbiota. The results revealed that nitrogen played a pivotal role in bacterial community changes.

### The connection among various bacteria

**Figure 5:**
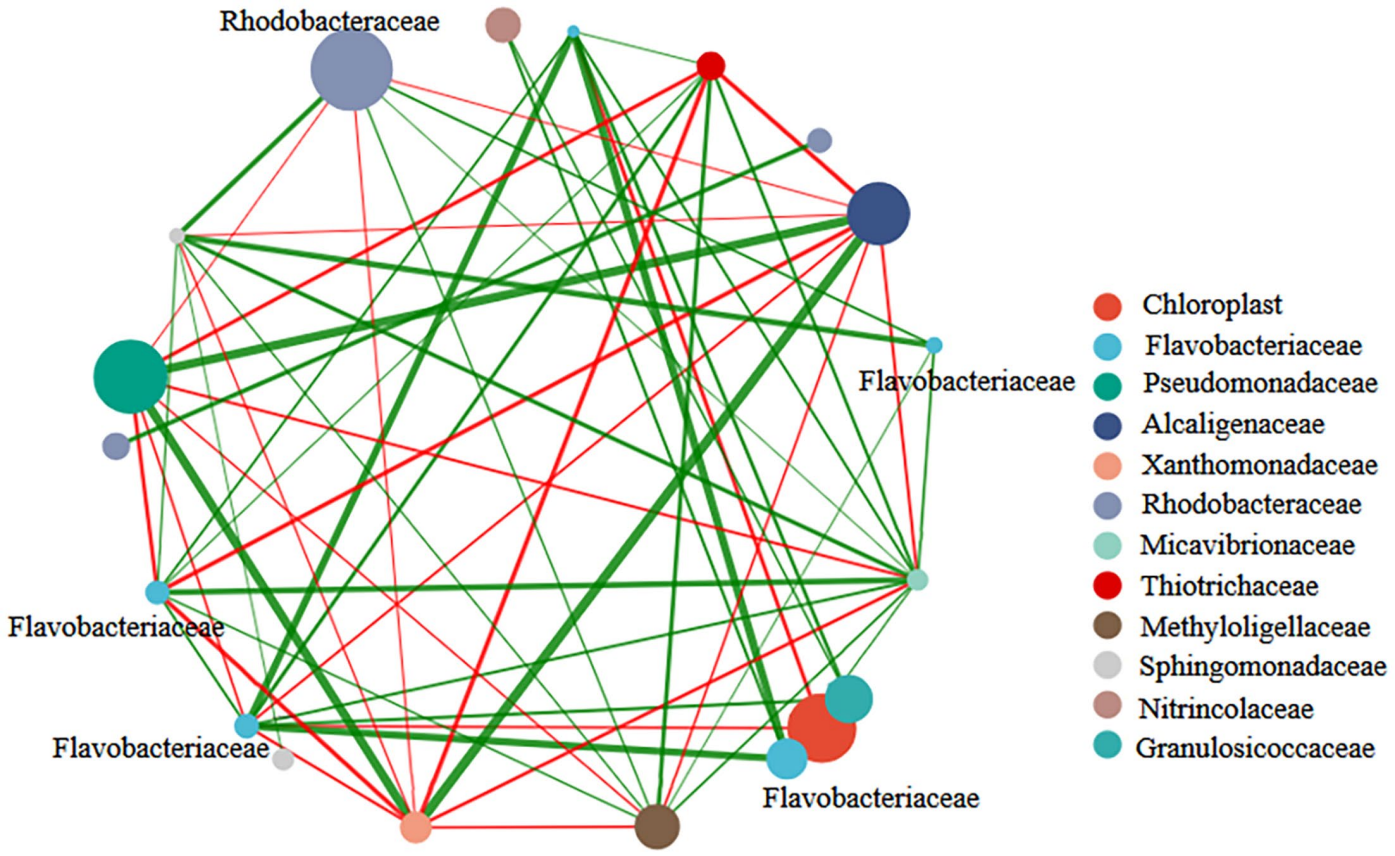
Correlation network diagram of bacterial genus levels in samples treated with different nitrogen concentration.

**Figure 6:**
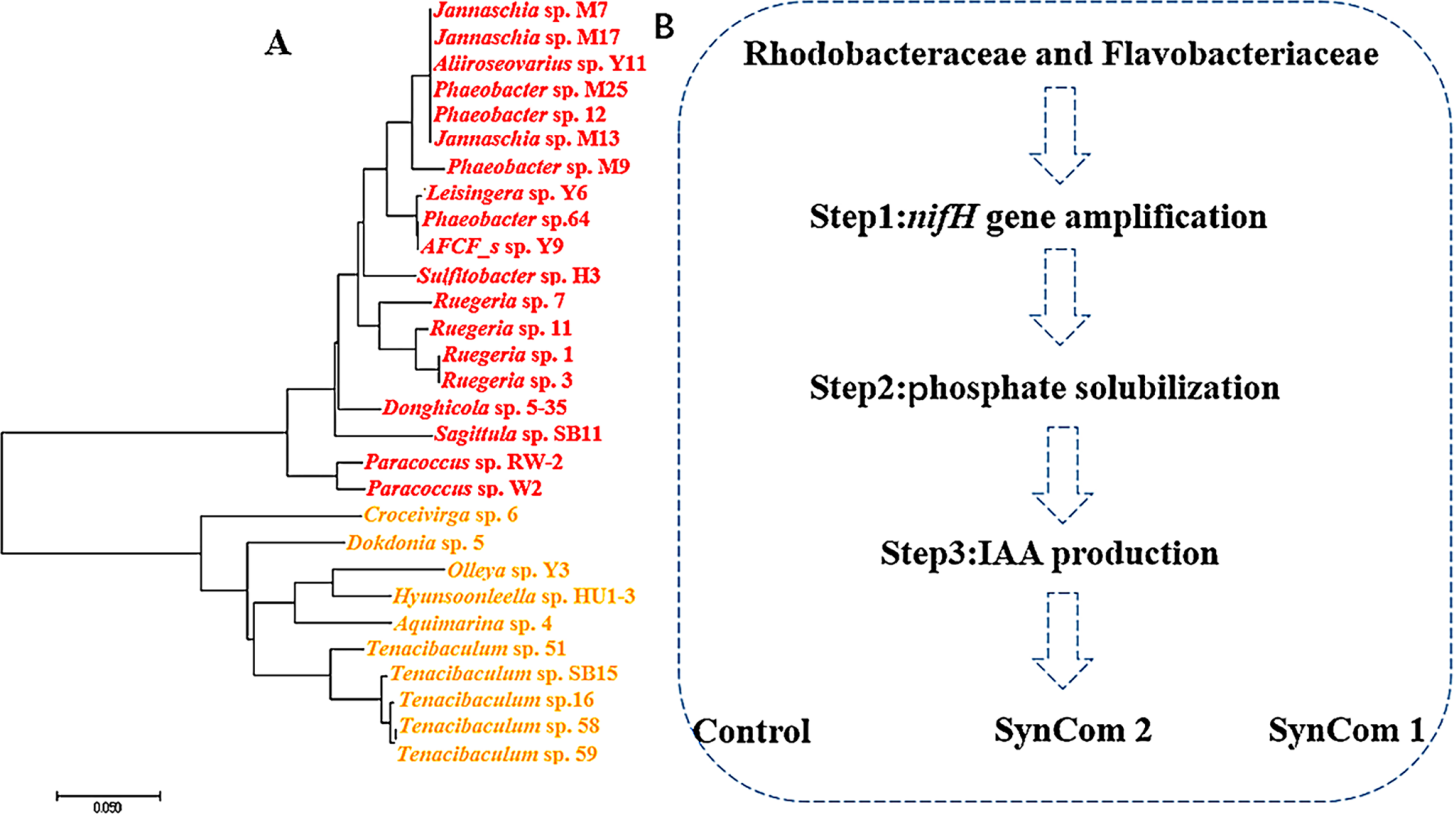
Rhodobacteraceae and Flavobacteriaceae stains **(A)** phylogenetic analysis and **(B)** functional screening of strains.

The network was constructed to understand the relationship among bacterial communities during the growth of *U. fasciata*. The nodes in the network were the top 13 species on the surface of *U. fasciata* with the highest total abundance (Figure 5). The strong network presence of Rhodobacteraceae might indicate its importance in stress resilience and adaptability within fluctuating environmental conditions, possibly acting as a stabilizing force in dynamic ecosystems. The positive correlations associated with Flavobacteriaceae indicate potential relationships with other microbial taxa within the community, as detected by correlation analysis. The combination of both positive and negative correlations for Rhodobacteraceae, compared with the primarily positive correlations for Flavobacteriaceae, suggests that these families play complementary roles in shaping microbial diversity and supporting the community’s overall metabolic efficiency. The clustering coefficients of Rhodobacteraceae and Flavobacteriaceae in the network analysis were 0.76 and 0.50, respectively. The members of these families were determined as key nodes in the network analysis. Although this was not the highest in the network analysis, changes in bacterial abundance under different nitrogen concentrations (Figure 1) and the species of pure bacteria obtained were considered (Hu et al., 2016). Therefore, Rhodobacteraceae and Flavobacteriaceae served as the basis of synchronization, and their evolutionary relationship is illustrated in Figure 6.

### Functional screening of *Ulva fasciata* surface-associated beneficial microbes

As mentioned earlier, Rhodobacteraceae and Flavobacteriaceae were selected as potential nitrogen-fixing strains. A total of 29 strains belonging to the families Rhodobacteraceae and Flavobacteriaceae were isolated from the surface of *U. fasciata*. Among them, 12 strains had *nifH*, 15 produced IAA, and 10 acquired Pi from insoluble phosphate (Pi) (Table 2). Some strains had two or three of these potential functions. A total of five strains (including *Ruegeria atlantica* 11, *Donghicola eburneus* 5–35, *Aliiroseovarius crassostreae* Y11, *Hyunsoonleella ulvae* HU1-3, and *Croceivirga radicis* 6) with high IAA and Pi productivity and the presence of *nifH* genes were selected. Four (removing *H. ulvae* HU1-3) were combined into the SynCom2 and co-cultured with WSB *U. fasciata* to determine the effects of nitrogen fixation and plant growth promotion.

**Table 2:** Phosphorus solubilizing capacity, IAA production capacity and *nifH* gene amplification results of Rhodobacaceae and Flavobacteraceae strains.

Rhodobacaceae and Flavobacteraceae strains	Phosphorus solubilizing capacity	IAA production capacity	*nifH* gene
*Ruegeria denitrificans* 1	−2.03 ± 0.23	**186.01 ± 3.66**	
*Ruegeria CP045384 s* 3	3.32 ± 4.67	10.67 ± 1.97	
*Ruegeria CP000377 s* 7	**102.73 ± 16.22**	**170.31 ± 6.05**	
*Ruegeria atlantica* 11	**90.11 ± 14.10**	**157.46 ± 3.99**	√
*Phaeobacter gallaeciensis* 64	15.68 ± 1.52	84.19 ± 6.64	
*Phaeobacter inhibens* 12	−1.90 ± 0.12	24.01 ± 0.58	√
*Sagittula stellata* SB11	8.48 ± 1.16	**156.01 ± 4.40**	√
*AFCFs* Y9	4.72 ± 0.28	78.92 ± 4.54	
*Leisingera daeponensis* Y6	1.56 ± 0.73	12.73 ± 13.62	
*Phaeobacter piscinae* M25	**154.23 ± 1.26**	**132.79 ± 1.03**	
*Pseudovibrio hongkongensis* H3	10.49 ± 2.92	**130.55 ± 4.10**	√
*Paracoccus seriniphilus* RW-2	11.33 ± 4.06	24.25 ± 5.50	√
*Paracoccus aquimaris* W2	**115.33 ± 11.34**	**188.37 ± 0.82**	
*Donghicola eburneus* 5–35	**88.15 ± 4.17**	**176.01 ± 9.86**	√
*Aliiroseovarius crassostreae* Y11	**97.82 ± 7.16**	**155.88 ± 4.21**	√
*Aquimarina spongiae* 4	**131.23 ± 0.62**	37.22 ± 1.09	√
*Dokdonia donghaensiss* 5	30.01 ± 0.69	24.25 ± 0.69	
*Croceivirga radicis* 6	**136.80 ± 3.88**	**165.28 ± 4.94**	√
*Tenacibaculum skagerrakense* 16	**108.53 ± 6.83**	**243.40 ± 10.55**	
*Tenacibaculum geojense* 51	17.92 ± 0.65	12.98 ± 13.47	
*Tenacibaculum aestuarii* 58	7.77 ± 1.54	**142.43 ± 5.08**	√
*Tenacibaculum singaporense* 59	−2.00 ± 0.08	**186.49 ± 7.25**	
*Tenacibaculum skagerrakense* SB15	6.24 ± 1.20	−2.66 ± 0.38	√
*Olleya algicola* Y3	1.99 ± 1.00	22.67 ± 4.11	
*Phaeobacter inhibens* M13	1.08 ± 0.16	**166.98 ± 0.58**	
*Phaeobacter inhibens* M17	8.33 ± 0.58	64.07 ± 6.88	
*Phaeobacter inhibens* M9	0.32 ± 0.12	39.52 ± 3.11	
*Phaeobacter inhibens* M7	13.56 ± 12.78	24.43 ± 4.16	
*Hyunsoonleella* HU1-3	**213.98 ± 1.15**	**227.64 ± 0.82**	√
In total	10	15	12

Bold represents a significantly larger value.

### Evaluation of functional assemblages of microbial community

**Figure 7:**
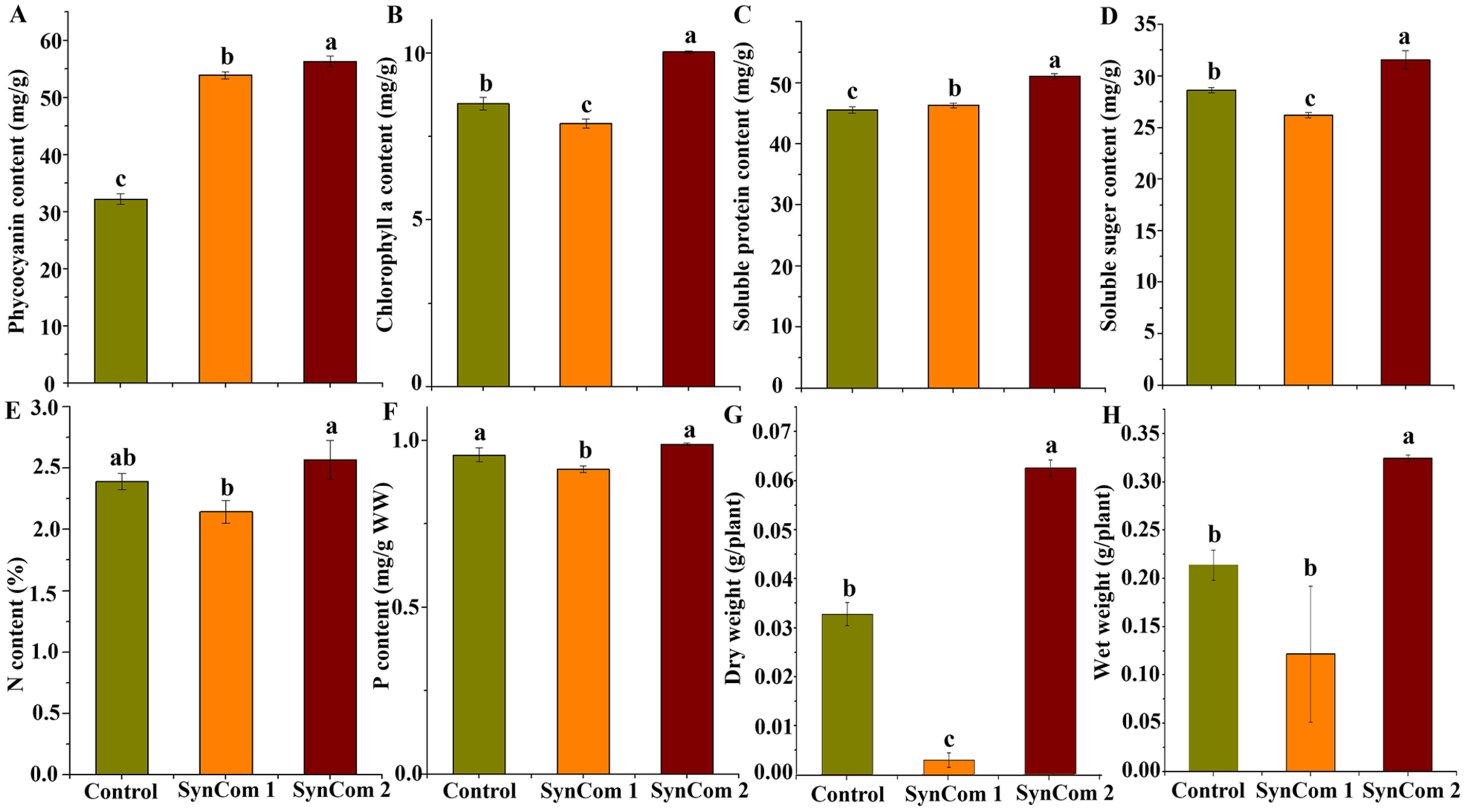
Changes of four physiological indices, N content, P content, dry weight and wet weight of sterile *U. fasciata* with two SynComs. **(A)** Phycocyanin **(B)** Chlorophyll a **(C)** Soluble protein **(D)** Soluble suger **(E)** N content **(F)** P content **(G)** Dry weight **(H)** Wet weight. The error bars are standard deviation; different letters on the error lines indicate significant differences between different treatments (α = 0.05).

**Figure 8:**
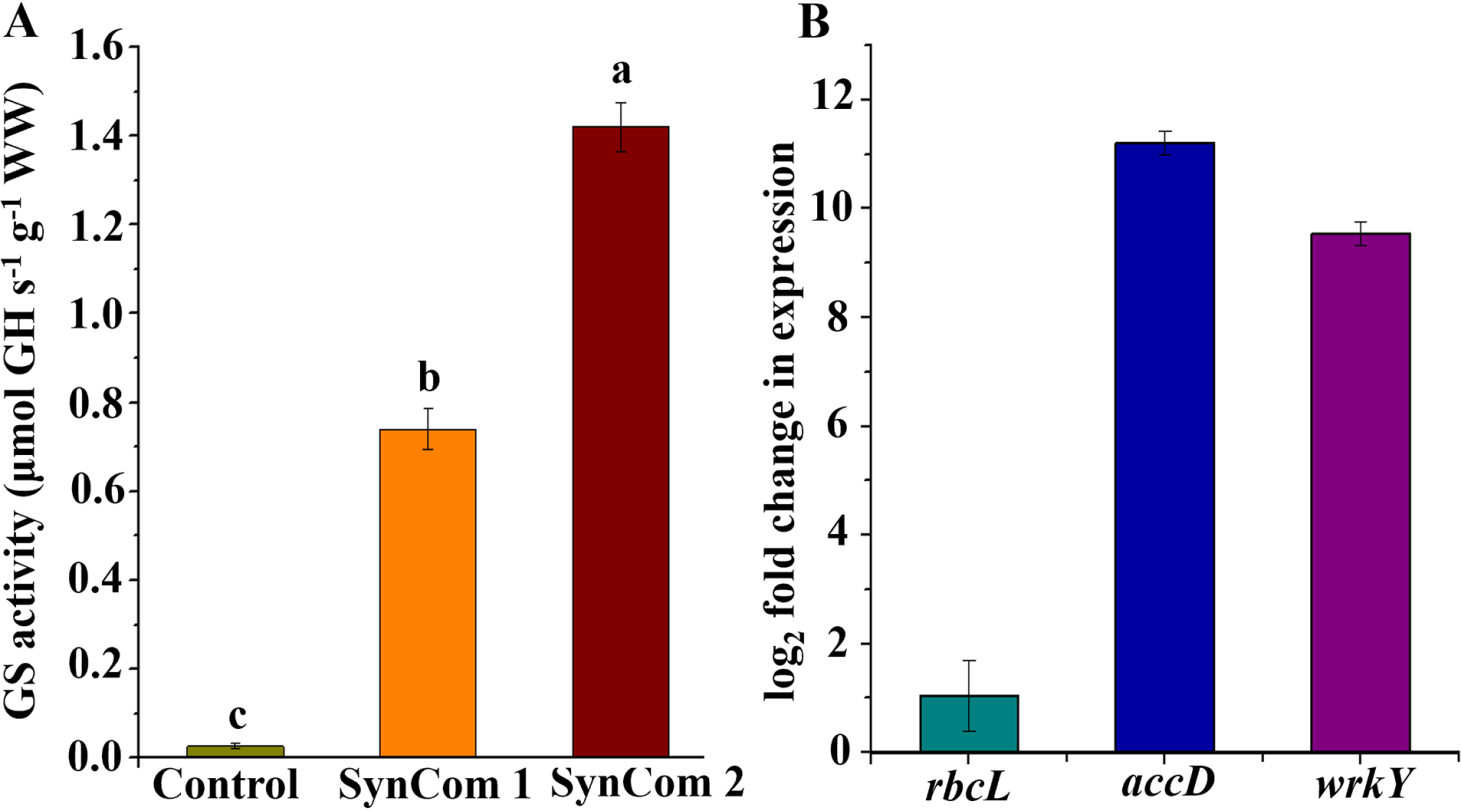
**(A)** Activity of glutamine synthetase with two SynComs and **(B)** *rbcL, accD* and *wrkY* gene expression multiples in SynCom2 compared with control.

In SynCom, it is generally accepted that the combined function of several microbial strains is more effective than that of a single strain (Falkowski, 1997). Two SynCom systems were structured, and SynCom1 was based on the potential functions of members of Rhodobacteraceae and Flavobacteriaceae. SynCom2 was established using four strains with beneficial functions. These SynCom systems were first co-cultured with WSB *U. fasciata* to examine the physiological parameters, including soluble sugar, protein, phycocyanin and Chlorophyll a, wet weight, dry weight, and nitrogen and phosphorus contents. The results indicated that, compared to the control, the physiological parameters of *U. fasciata*—including soluble sugar, soluble protein, phycocyanin, Chlorophyll a, wet weight, dry weight, and nitrogen and phosphorus contents—changed within the following ranges: −8.4–10.1%, 1.6–16.8%, 23.9–49.2%, −6.7–18.3%, −6.0–5.1%, −6.6–10.1%, −11.7–7.5%, and −4.4–3.3%, respectively (Figures 7A–H). In addition, two SynCom systems significantly increased glutamine synthesis activity, which was almost undetectable in the control group (Figure 8A). The superiority of SynCom2 over SynCom1 in facilitating nitrogen and phosphorus acquisition is evident from the experimental data, where SynCom2 promoted more efficient nutrient uptake, enhanced *U. fasciata* growth, and increased nitrogen fixation compared to SynCom1. Moreover, SynCom2’s stability was demonstrated by the successful identification of all four strains after the experiment, whereas identifying specific species in SynCom1 was challenging due to varying growth rates of strains.

### Regulation of vital transcriptional genes in *Ulva fasciata* by SynCom2

Since SynCom2 was highly correlated with the growth of *U. fasciata*, the *U. fasciata* co-cultured with SynCom2 was further analyzed by RT-qPCR to investigate the underlying mechanisms of several key genes associated with nitrogen fixation and plant growth. The expression levels of the three key genes involved in photosynthesis (*rbcL*), lipid biosynthesis (*accD*), and growth hormone precursor (*wrkY*) pathways were determined by adding *U. fasciata* to SynCom2 in nitrogen-free conditions. The increase in gene expression observed in SynCom2 compared to the control group refers to the upregulation of functional genes involved in nitrogen fixation, phosphorus acquisition, and other metabolic processes that support the growth of *U. fasciata*. These functional genes were activated to varying degrees in response to co-culturing with SynCom2, leading to enhanced nutrient uptake and growth efficiency. The expression levels of *rbcL*, *accD*, and *wrkY* were elevated by 1.04-, 11.21-, and 9.54-fold, respectively.

## Discussion

Nitrogen is a key element for life and a limiting factor for marine productivity in numerous global ocean regions (Glaze et al., 2022). Nitrogen in the ocean has different forms, including ammonium (NH_4_^+^) and nitrate (NO_3_^−^). In recent *years*, studies on microbial communities in marine nitrogen cycling have notably received scholars’ attention (Lin et al., 2021). In this study, the bacterial species on the surface of *U. fasciata* under varying nitrogen concentrations were determined by high-throughput sequencing. The results indicated that the microbiota on the surface of *U. fasciata* changed with the growth time and stabilized at the late growth stage (Figure 1). Under typical nitrogen levels used in this experiment or commonly found in natural environments, nitrogen positively affects Flavobacteriaceae, Thiotrichaceae, Rhodobacteraceae, Nitrincolaceae, Methyloligellaceae, and other bacteria (Suliasih and Widawati, 2005; Madhaiyan et al., 2010; Ugarelli et al., 2018). As these bacteria are nitrogen-fixing communities, it is noteworthy that the relative abundance rates of these bacteria increased when cultivated under low nitrogen concentrations. Consistent with previous studies (Preena et al., 2018), indicating that Rhodobacteraceae members are nitrogen-fixing bacteria crucial for host physiology and health, our findings revealed a decrease in the abundance of these bacteria at higher nitrogen concentrations. Previous studies have demonstrated that the composition of epiphytic microorganisms from algae is mainly affected by ambient environmental factors (Burke et al., 2011; Singh and Reddy, 2016). According to the results of PCoA and RDA, our data also showed that nitrogen concentration in the environment greatly impacted the surface bacterial community of *U. fasciata* (Figures 3, 4).

A core microbiome is a group of shared members of microbial communities from similar habitats (Shade and Handelsman, 2012). Therefore, discovering the core microbiome is essential to understanding the composition of complex microbial assemblages. In this study, the core microbiota were defined not only by their relative abundance but also by their ecological roles and interactions with the host *U. fasciata* and environmental factors, such as varying nitrogen concentrations. Despite changes in nitrogen levels, the core microbiome on the surface of *U. fasciata* remained consistent. By characterizing epiphytic bacteria associated with *Ulva* (Chlorophyta), Lachnit et al. (2011) demonstrated that each algae contains species-specific and time-adapted bacterial communities on its surface. Marzinelli et al. (2015) proposed that biological and abiotic factors affect bacterial communities associated with macroalgae. The variation in nitrogen concentrations in marine environments fundamentally alters the microbial landscape, as demonstrated by the shifts in the surface microbiota of *U. fasciata*. The increase in nitrogen-fixing bacteria, such as Rhodobacteraceae and Flavobacteriaceae, under low nitrogen conditions supports the principle that nutrient limitation favors organisms capable of utilizing alternative nutrient sources, such as atmospheric nitrogen. This adaptation highlights the crucial role of these bacteria in nitrogen cycling and the broader marine nitrogen budget. Despite variations in nitrogen level, the core microbiome of *U. fasciata* remains relatively stable. This stability may indicate a co-evolved relationship between the algae and its epiphytic microbiota, where certain bacterial species are essential for maintaining algal health regardless of nutrient availability. These core bacteria may provide critical ecosystem services, such as nitrogen fixation, phosphorus solubilization, and pathogen suppression, necessary for algal growth across different environmental conditions (Thaweenut et al., 2011; Smercina et al., 2019).

Network analysis was used to determine the interaction among strains (Chun et al., 2020; Prashar et al., 2013). According to the results of PCoA and RDA, some genera of the Rhodobacteraceae family were essential nodes. Members of *Rhodobacteraceae* exhibited a strong positive correlation with other genera (indicated by the red line), leading to their identification, along with *Flavobacteriaceae*, as strains with potential functional roles within the community. The positive correlations between Rhodobacteraceae and other bacterial genera suggest these families act as keystone species in the microbial network. Their ability to fix nitrogen and promote nutrient cycling likely supports the growth of other associated microorganisms, further enhancing the resilience and functionality of the microbial community. This interconnectedness reflects the broader ecological principle that microbial communities are structured by nutrient availability and species interactions that promote collective stability. The bioinformatic analyses highlighted the abundance of Rhodobacteraceae and Flavobacteriaceae. They pointed to their functional potential in nitrogen cycling, as inferred from the significant associations in Spearman’s correlation network and RDA results.

Additionally, the Shannon index and other alpha-diversity metrics revealed that environments enriched in nitrogen displayed a higher diversity of nitrogen-cycling bacteria, with Rhodobacteraceae and Flavobacteriaceae consistently contributing to this microbial richness. Given their strong statistical association with nitrogen levels and significant differential abundance identified through ANCOM, these families were further tested in synthetic bacterial communities to validate Sheng Xin’s hypothesis on nitrogen fixation. The experimental results aligned with the bioinformatic predictions, confirming the contribution of these bacterial groups to nitrogen assimilation and *Ulva* growth.

Recent studies have demonstrated that plant-associated microbiota can influence their host’s disease resistance, nutritional status, and growth rates (Thaweenut et al., 2011). The isolation of these beneficial microorganisms is of great importance in promoting the growth of plants, including algae (Ferranti and Delwiche, 2024). Notably, the *nifH* gene encodes a nitrogenase enzyme that plays a crucial role in nitrogen fixation by facilitating the production and regulation of nitrogenase (Wang et al., 2021). Hence, microorganisms carrying the *nifH* gene could be utilized as candidate bacteria for nitrogen fixation in plants. This study identified potential growth-promoting strains by obtaining microorganisms capable of phosphorus acquisition.

Using the above-described screening methods, 5 of the 29 strains containing the *nifH* gene were identified to have high IAA and Pi production. A co-culture experiment evaluated the effects of the four strains in the SynCom2 on the growth of *U. fasciata*. In another study, *H. ulvae* HU1-3 alone significantly promoted *U. fasciata* biomass accumulation. However, the SynCom comprising five strains, including HU1-3, did not enhance biomass. The effect of the SynCom with 29 strains was nearly identical to that of the SynCom with 28 strains after excluding *H. ulvae* HU1-3 (results not shown). We selected a mixture of 29 strains (SynCom1) as a positive control to demonstrate the effectiveness of the synthetic method. These four bacteria significantly increased the contents of soluble sugar, protein, phycocyanin and Chlorophyll a, wet weight, dry weight, nitrogen, phosphorus, and other physiological parameters of *U. fasciata* (Figures 7A–H). The results indicated that the four bacterial strains had health benefits to the *U. fasciata*. The experimental results also demonstrated that high-throughput sequencing was used to predict potential functional bacteria in this study. Then, joint functional screening was found to be an effective method of identifying potentially beneficial microorganisms. It should be noted that other potentially helpful functions, such as Acc deaminase activity and siderophore production, are also noteworthy (Bruto et al., 2014).

In the SynComs development, each strain’s function was considered. Based on these functions, the results indicated that SynCom2 could significantly improve plant growth and nitrogen and phosphorus acquisition compared with SynCom1. The combination of functions of the SynCom systems adopted in this study is an effective strategy to promote plant growth and nutrient acquisition. The significant improvement in growth parameters and nutrient acquisition by *U. fasciata* when treated with SynCom2 suggests that microbial functional diversity is crucial in promoting plant health. The specific activation of essential genes related to photosynthesis (*rbcL*), lipid biosynthesis (*accD*), and auxin production (*wrkY*) by the SynCom2 community indicates that these bacteria do more supply nutrients—they actively regulate host gene expression, directly influencing metabolic pathways and developmental processes.

Then, RT-qPCR was carried out using SynCom2 to determine whether the expression levels of some key genes were significantly affected by the colonization of the microbial community (Figure 8B). It was reported that the *rbcL* gene was associated with chlorophyll content (Ohmiya et al., 2014). Accd gene could regulate the activity of acetyl-CoA carboxylase and be involved in the fatty acid biosynthesis pathway (Kumar et al., 2017). However, the *wrkY* gene could regulate plant development and produce auxin precursors, ultimately emerging beneficial for the plant growth (Yuan et al., 2017). The effect of SynCom2 on *U. fasciata* growth was further verified by quantifying the expression levels of key genes involved in photosynthesis (*rbcL*), lipid biosynthesis (*accD*) and plant development regulators (*wrkY*) pathways. *In addition*, the results of the present study revealed that IAA genes were activated by the SynCom2. These findings indicated that the SynCom2 not only improves the photosynthetic capacity of *U. fasciata* leaves, but also activates plant growth promotion signals and increases bioaccumulation. However, further investigation is required to explore how SynCom2 affects changes in *U. fasciata*. The results suggested a close genetic relationship between *U. fasciata* and its epiphytic microorganisms, and microbes in SynCom2 could regulate the transcription of genes related to host growth. Future research will concentrate on uncovering the precise molecular mechanisms by which these bacterial communities influence algal gene expression and development. Additionally, exploring the potential functions of other microbial activities, such as ACC deaminase activity and siderophore production, may reveal further insights into how these epiphytic bacteria contribute to nutrient acquisition and stress tolerance in *U. fasciata*.

## Conclusion

This study systematically examined the bacterial community associated with *U. fasciata* under varying nitrogen concentrations, revealing significant changes in the relative abundance of key bacterial families, including Rhodobacteraceae and Flavobacteriaceae. Network analysis identified critical interactions in the microbial communities, and functional screening highlighted nitrogen-fixing strains as beneficial to *U. fasciata* growth. The establishment of SynCom systems, particularly SynCom2, demonstrated superior performance in promoting nitrogen fixation, nutrient acquisition, and enhancing plant growth under nitrogen-free conditions. Expression analysis of key genes in *U. fasciata* further validated these effects, showing increased activity in pathways related to photosynthesis, lipid biosynthesis, and hormone production. The findings provide valuable insights into how nitrogen concentrations shape the surface microbiota of *U. fasciata*, with potential applications in microbiome-based strategies to enhance plant growth. The development of SynCom systems presents an innovative approach to improve nitrogen assimilation and nutrient uptake, not only in *U. fasciata*, but also potentially in other aquatic plants. These results could lead to the advancement of sustainable aquaculture practices and broader ecological applications, where microbial communities are leveraged to optimize plant growth and nutrient cycling. Future studies will explore the application of SynCom systems across various plant species and assess their long-term ecological influences on different aquatic environments.

## Data Availability

Bacterial 16S rRNA gene sequencing data were uploaded to the NCBI SRA database, with accession number of PRJNA772012.
