# Supplementary material for: Functional assembly of surface microbiota of Ulva fasciata improves nutrient absorption efficiency and growth
Source: Front Microbiol. 2024 Dec 3;15:1476073. doi: 10.3389/fmicb.2024.1476073 (PMC11649579; doi:10.3389/fmicb.2024.1476073)
Supplement: Supplementary file 1 [file Table_1.docx]

**Supplemental Table S1.** Supplemental file 1 is a Table that shows primers used for the gene expression of rbcL, accD and genes using real-time PCR (RT-qPCR).

| Name | Gene | Prmer | |
| --- | --- | --- | --- |
| Internal control | actin*-*F | ATCGCAGCCTTCAACTAG |  |
|  | actin*-*R | ATGTCTGTCCACCTTCAC |  |
| Lipid biosynthesis | *accD-*F | TTTCCTTTCTCCTTCTGGTG |  |
|  | *accD-*R | CACCACCACTTGTTGGAGAA |  |
| Photosynthesis | *rbcL-*F | CTTTCCAAGGTCCTCCTCAC |  |
|  | *rbcL-*R | TCTCTCCAACGCATAAATGG |  |
| Growth hormone precursor | *wrky-*F | CACCAGGACATATACACA |  |
|  | *wrky-*F | AACACCAGCACTTATCTA |  |
